# AlphaGenome Enhances Personal Gene Expression Prediction but Retains Key Limitations

**DOI:** 10.21203/rs.3.rs-7520707/v1

**Published:** 2025-09-09

**Authors:** Li Shen

**Affiliations:** 1 Department of Artificial Intelligence and Human Health, Icahn School of Medicine at Mount Sinai, New York, USA.; 2 Department of Neuroscience, Icahn School of Medicine at Mount Sinai, New York, USA.

**Keywords:** gene expression, deep learning, genome AI model, personalized prediction

## Abstract

In recent years, numerous genome AI models have been developed to elucidate the relationship between DNA sequence and gene expression. However, these models have faced criticism for their limited accuracy in predicting individual-specific gene expression. AlphaGenome, the current state-of-the-art in genome AI, achieves exceptional performance across a range of sequence-based predictive tasks, but its utility for personal expression prediction has not yet been assessed. In this study, we evaluate AlphaGenome’s ability to predict personal gene expression and find that it significantly outperforms its predecessor. Using GTEx data, AlphaGenome improves the prediction of expression direction over Enformer, achieving an odds ratio of 3.0. In some cases, it even reverses previously observed negative correlations into positive ones. Moreover, AlphaGenome demonstrates improved performance for genes with known nonlinear sequence-expression relationships, though it uncovers mechanisms distinct from those identified by tree-based models.

## Introduction

Understanding the relationship between genome sequences and molecular phenotypes–such as chromatin modifications, DNA accessibility, and gene expression–has long been a central focus in genomics. Among these phenotypes, gene expression is arguably the most extensively studied due to its strong associations with health and disease [1]. Over the past few decades, investigations into the functional link between sequence and expression have largely fallen under the scope of expression quantitative trait locus (eQTL) analysis [2]. Traditional machine learning approaches, including penalized linear regression [3–5] and random forests [6], have been commonly employed in this context.

More recently, deep learning-based genome AI models have emerged as powerful alternatives to traditional methods [7–13], demonstrating remarkable performance in predicting a wide range of molecular phenotypes across previously unseen genomic regions. Among these, Enformer [11] represents a landmark advancement, combining convolutional and transformer layers to capture long-range interactions and outperform prior approaches. At the time of its release, Enformer featured significantly more parameters than its predecessors and required substantially greater computational resources for training. Building on this foundation, AlphaGenome [13] is currently the state-of-the-art (SOTA) genome AI model. It employs a 1 Mb context window and delivers predictions at single-basepair resolution, achieving substantial improvements over existing methods across multiple predictive tasks.

The impressive performance of genome AI models has sparked growing interest in their potential to predict molecular phenotypes–particularly gene expression–at the individual level. Such capabilities hold significant promise for applications in drug target discovery and precision medicine. However, early investigations have yielded disappointing results: many of Enformer’s predictions were negatively correlated with observed expression levels [14, 15]. In response, researchers have attempted to fine-tune Enformer and similar models using individual-specific data, yielding modest but encouraging improvements [16, 17].

This study aims to evaluate the potential of AlphaGenome for predicting personal gene expression. Although AlphaGenome was not explicitly trained on individual-level data, its enhanced network architecture and expanded training set make it reasonable to hypothesize that it may outperform its predecessors. To assess its capabilities and limitations, we compare AlphaGenome against its predecessor, Enformer, as well as two classic machine learning methods: Elastic Net and Random Forest. A key advantage of deep learning-based models over traditional approaches lies in their ability to capture complex, nonlinear relationships within data. To investigate this strength, we focus on a subset of genes for which the sequence-expression relationships are likely nonlinear. Additionally, a case study is conducted to examine the marginal effects of genetic variants on AlphaGenome’s predictions.

## Results

### AlphaGenome Significantly Outperforms Its Predecessor in Predicting Personal Gene Expression

The GTEx database [18], which includes RNA-seq data from 953 individuals across 50 tissues, was used as a benchmark to evaluate each model’s ability to predict personal gene expression. The normalized expression matrix, downloaded from the GTEx website, contains 42,372 genes. After filtering for sparsity, 377,857 gene-tissue pairs remained for model training and evaluation. Both Elastic Net and Random Forest were trained and evaluated separately for each gene using nested 10-fold cross-validation. Based on the results from Elastic Net, 300 genes were randomly selected to span a range of coefficients of determination (R2) across tissues–from negative values to greater than 0.5. AlphaGenome was originally trained to predict population-averaged expression levels for all GTEx tissues; thus, its predictions for individual sequences on the selected 300 genes were used directly. Since Enformer does not natively output GTEx tissue predictions, we followed AlphaGenome’s approach: Enformer’s frozen embeddings were used to train predictors for population-averaged expression across all GTEx tissues, and these predictors were then applied to individual sequences. For fair comparison across models, Pearson correlation was used as the evaluation metric, as it does not require scale-matching between predicted and observed values.

**Fig. 1:**
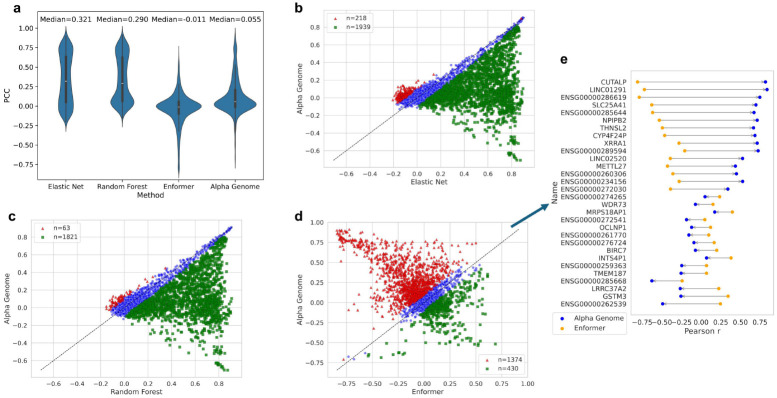
Accuracy of personal gene expression prediction for four comparing methods. **a** Violin plots of Pearson correlations of the four methods across all gene-tissue pairs. **b-d** Scatter plots of AlphaGenome vs. Elastic Net, Random Forest and Enformer. Red triangles represent gene-tissue pairs that AlphaGenome is significantly better, while green squares represent gene-tissue pairs that other methods are significantly better. The statistical significance is determined from 1000 bootstrap samples. **e** Dumbbell plots of the top 15 genes from AlphaGenome and Enformer based on the difference of Pearson correlation.

Figure 1a presents the distribution of Pearson correlation coefficients across all gene-tissue pairs for the four methods compared. As expected, both AlphaGenome and Enformer exhibit lower correlations than Elastic Net and Random Forest, as neither deep learning model was trained on individual-level data. Notably, AlphaGenome outperforms Enformer on average, with a median correlation higher by 0.07 and an even greater difference at the upper quartile. Among all gene-tissue pairs, AlphaGenome yields 2,459 positive and 971 negative correlations, while Enformer yields 1,557 positive and 1,873 negative correlations. This is particularly striking given that AlphaGenome was not trained on personal expression data.

To further assess their relative performance, head-to-head comparisons were conducted between AlphaGenome and each of the other three methods across all gene-tissue pairs. AlphaGenome significantly outperforms Elastic Net in 218 gene-tissue pairs (Figure 1b) and Random Forest in 63 gene-tissue pairs (Figure 1c). When compared to Enformer, AlphaGenome shows a clear advantage: 1,374 gene-tissue pairs have significantly higher correlations than Enformer, while only 430 favor Enformer, resulting in a winning ratio of 3.2.

To gain a more intuitive understanding of AlphaGenome’s improvement over Enformer, the top 15 genes from each model–ranked by the difference in Pearson correlation in lung tissue–are shown in Figure 1e. For AlphaGenome, the top-performing genes exhibit dramatic reversals in correlation direction; for example, CUTALP improves from a correlation of −0.81 to +0.82. In contrast, the improvements among Enformer’s top genes are comparatively modest. These results highlight AlphaGenome’s substantial advantage in predicting personal gene expression, despite not being explicitly trained for this task.

### AlphaGenome Captures Nonlinear Dependencies with Mechanisms Distinct from Tree-Based Methods

**Fig. 2:**
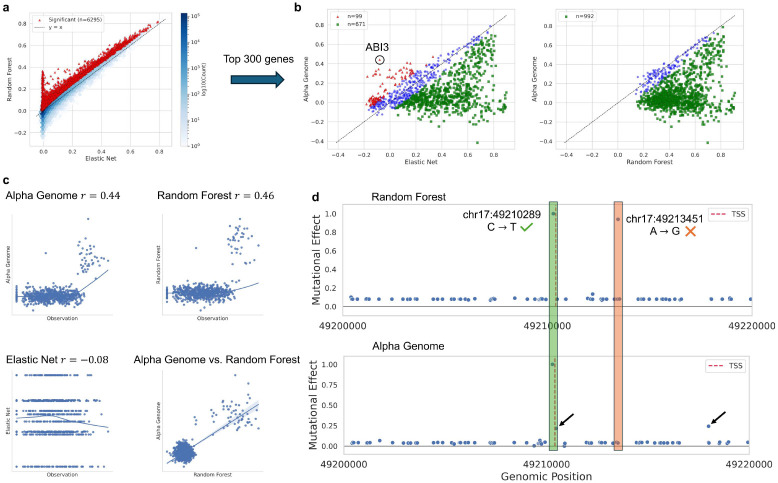
AlphaGenome vs. Random Forest and Elastic Net in nonlinearity-filtered gene-tissue pairs. **a** Scatter plot of Random Forest vs. Elastic Net to identify nonlinear gene-tissue pairs. **b** Scatter plots of AlphaGenome vs. Elastic Net and Random Forest for top 300 nonlinear genes. **c** Scatter plots of the comparing model’s predictions vs. observation. **d** Marginal effects of genetic mutations for Random Forest and AlphaGenome in a 20 Kb region around the transcriptional start site of ABI3.

Random Forest compares more favorably than Elastic Net against AlphaGenome, with fewer gene-tissue pair losses (63 vs. 218). This supports the hypothesis that AlphaGenome is more likely to outperform Elastic Net when the sequence-expression relationship is nonlinear. Since Random Forest can capture such nonlinear patterns, it reduces AlphaGenome’s relative advantage. Therefore, Random Forest was used as a nonlinearity filter to identify 6,295 gene-tissue pairs where it significantly outperformed Elastic Net (Figure 2a), representing approximately 1.7% of all pairs.

While it is possible that other gene-tissue pairs exhibit nonlinear relationships that Random Forest fails to capture–but that AlphaGenome could–this is considered unlikely, and running AlphaGenome across all 377,857 gene-tissue pairs is computationally impractical. From the filtered set, the top 300 genes (based on differences in correlation with Elastic Net) were selected to compare AlphaGenome with Elastic Net and Random Forest. AlphaGenome outperformed Elastic Net in 99 gene-tissue pairs but did not outperform Random Forest in any (Figure 2b). This outcome may be partially biased, as the selected gene-tissue pairs were pre-filtered based on Random Forest’s performance.

To gain deeper insight into how AlphaGenome outperforms Elastic Net on genes with nonlinear relationships, the top-ranking gene, ABI3, was selected as a case study. Model predictions in cultured fibroblast cells were compared to observed expression values across 953 individuals, as shown in Figure 2c. Both AlphaGenome and Random Forest achieved comparable correlations with the observed data (0.44–0.46), whereas Elastic Net produced a slightly negative correlation. Despite the similarity in overall correlation, AlphaGenome and Random Forest made distinct predictions: while both successfully classified individuals into high- and low-expression groups, their predictions within each group showed minimal correlation, suggesting that the two models capture different underlying patterns.

In silico mutagenesis was performed on all identified variants within a 1 Mb window centered on the transcriptional start site (TSS) of ABI3 to assess their marginal effects on predictions from Random Forest and AlphaGenome. A 20 Kb region surrounding the TSS–where the strongest effects were observed–is shown in Figure 2d. Both models identified a C → T mutation at chr17:49,210,289 as having the most significant effect. However, the two models diverged in their assessment of other variants. Random Forest identified an A → G mutation at chr17:49,213,451 as important, while AlphaGenome showed minimal response to this variant. Conversely, AlphaGenome highlighted two other mutations with moderate effects (normalized impact ≈ 0.2) that were not detected by Random Forest. These differences suggest that Random Forest and AlphaGenome capture distinct nonlinear relationships between sequence variation and gene expression for the same gene.

## Discussion

### Is bigger always better?

For researchers in the field of large language models (LLMs), the answer is often a resounding yes, as the scaling law has consistently held true [19]. We are currently witnessing the rise of extreme-scale LLMs–such as GPT-4 [20] and Grok 4 [21]–that continue to grow in size and capability. A similar trend may be unfolding in genomics, as the declining cost of sequencing paves the way for personal genomes to be generated at unprecedented scale [22].

AlphaGenome, with its 1 Mb context window and single-basepair resolution, represents the current state-of-the-art in genome AI. It has demonstrated exceptional accuracy in predicting molecular phenotypes across multiple modalities using the reference genome. As shown in this study, AlphaGenome also improves the prediction of individual-level gene expression–despite never being trained on personal data. This performance gain likely stems from advances in both model architecture and training data: single-basepair resolution increases sensitivity to SNVs and indels, the most common types of genetic variation; the expanded context window enables the model to integrate long-range regulatory interactions; and multimodal training offers a more holistic understanding of the transcriptional regulatory code.

Despite its advancements, AlphaGenome’s performance still lags behind that of classic machine learning models trained directly on personal-level data. Currently, training AlphaGenome on such data is infeasible, as DeepMind has only released APIs for model inference and explicitly prohibits fine-tuning AlphaGenome outputs for downstream tasks [23]. However, it is anticipated that future models of this kind will be developed, enabling deeper insights into the relationship between genome sequences and molecular phenotypes–not only across genomic regions, but also at the individual level.

## Methods

### Data Preprocessing

For RNA-seq data, normalized expression values (V10) were downloaded from the GTEx website, with no additional processing applied. For each gene, a tissue was excluded if more than 50% of expression values were missing. For personal genomes, phased whole-genome sequencing data (V9) were used. In non-deep learning models, genetic variants from the two haplotypes were treated as separate features and concatenated. Both SNVs and indels were included, and variants with a minor allele frequency below 1% were filtered out. For AlphaGenome and Enformer, both haplotype sequences were used as input, and the resulting outputs were averaged to produce final predictions.

### Use AlphaGenome and Enformer for Personal Expression Prediction

For AlphaGenome, a 1 Mb window centered on the transcriptional start site (TSS) of each gene was used as input. The model outputs predictions over the same window at single-basepair resolution. For each gene, predicted expression values from the TSS to the transcriptional end site (TES) were averaged to represent that gene’s expression level. AlphaGenome’s half-window size of 500 Kb is sufficient to span the full gene body (TSS to TES) for the vast majority of genes; only 422 genes–approximately 0.7% of all GTEx genes–exceed 500 Kb in length.

Enformer, by contrast, requires training to predict GTEx tissue-level expression. The same train-validation-test split as used in the Borzoi [12] model was adopted, where each GTEx gene was assigned to a set based on the genomic region closest to its TSS. A 196,608 bp window centered on the TSS was extracted from the reference genome and used as input to the pretrained Enformer to obtain an embedding. As with AlphaGenome, predicted values from TSS to TES were averaged to represent gene-level expression. The target values were defined as the average expression across all GTEx individuals for each tissue.

The position-averaged embedding vectors were then used to train a Ridge regressor to predict tissue-specific expression levels. The regularization strength was selected from 30 evenly spaced values in log space between 1 and 5. The optimal model was chosen based on R2 performance on the validation set. For personal-level expression prediction, individual genome sequences were fed into Enformer to generate embeddings, which were then input into the trained Ridge regressor to produce tissue-specific expression predictions.

### Choosing 300 Genes that Span Different R2 Values

The R2 values from 10-fold cross-validation using Elastic Net were used as a reference to assess gene predictability. Genes were grouped into 10 bins based on their R2 values across tissues: (−∞,0], (0,0.01], (0.01,0.02], (0.02,0.05], (0.05,0.1], (0.1,0.2], (0.2,0.3], (0.3,0.4], (0.4,0.5], and (0.5,1]. From each bin, 30 genes were randomly selected, resulting in a set of 300 genes that span a wide range of predictability levels.

### *In silico* mutagenesis

*In silico* mutagenesis (ISM) was performed to estimate the marginal effect of individual genetic mutations on AlphaGenome’s output. Each mutation was introduced into a reference sequence spanning 1 Mb centered on the TSS, and the resulting predicted expression was compared to the prediction from the unmodified reference sequence. The difference between these two predictions was taken as the mutation’s effect.

For Random Forest, the reference expression was obtained using a feature vector of all zeros. Each mutation was then individually activated (set to one) to compute the corresponding alternative expression. To enable direct comparison between AlphaGenome and Random Forest, the marginal effects for all mutations were rescaled to the [0, 1] range.
